# Invasive pneumococcal disease unmasks monoclonal immunoglobulins and antibody deficiencies: a multicenter prospective study in adults

**DOI:** 10.1038/s41598-026-61992-8

**Published:** 2026-07-24

**Authors:** Tor Härnqvist, Karin Bergman, Åsa Mellgren, Magnus Brink, Amanda Nilsson, Staffan Nilsson, Bengt Andersson, Rune Andersson, Anna Lundgren, Johanna Karlsson, Susann Skovbjerg

**Affiliations:** 1 Department of Infectious Diseases, Institute of Biomedicine, Sahlgrenska Academy, University of Gothenburg, Guldhedsgatan 10A, SE-413 46 Gothenburg, Sweden; 2 Department of Infectious Diseases, NU Hospital Group, Trollhättan, Region Västra Götaland Sweden; 3 Department of Infectious Diseases, Södra Älvsborg Hospital, Borås, Region Västra Götaland Sweden; 4 Department of Infectious Diseases, Sahlgrenska University Hospital, Gothenburg, Region Västra Götaland Sweden; 5 Department of Laboratory Medicine, Institute of Biomedicine, Sahlgrenska Academy, University of Gothenburg, Gothenburg, Sweden; 6 Department of Clinical Immunology and Transfusion Medicine, Sahlgrenska University Hospital, Gothenburg, Region Västra Götaland Sweden; 7 Department of Clinical Microbiology, Sahlgrenska University Hospital, Gothenburg, Region Västra Götaland Sweden; 8 Department of Microbiology and Immunology, Institute of Biomedicine, Sahlgrenska Academy, University of Gothenburg, Gothenburg, Sweden

**Keywords:** Invasive pneumococcal disease, Immune deficiency, Immunoglobulin, Hypogammaglobulinemia, Monoclonal gammopathy of undetermined significance, M protein, Multiple myeloma, Cancer, Diseases, Immunology, Medical research, Oncology

## Abstract

Immunoglobulins (Ig) are essential for protection against invading pneumococci but are not routinely analyzed in patients with invasive pneumococcal disease (IPD). In this prospective study, we assessed the prevalence of monoclonal Ig (M protein) and analyzed Ig concentrations in sera from 156 adult IPD patients (median age 70 years). Patients were sampled during acute infection (*n* = 153) and in the convalescence phase 2–4 months after acute infection (*n* = 76). Sixty-four sex- and age-matched individuals without IPD served as controls. During infection, M protein was detected in 22% (31/141) of patients without previously known hematological malignancy as compared with 5% of the controls (*p* = 0.002). Seven of these patients were subsequently diagnosed with a hematological malignancy, including multiple myeloma, Waldenstrom macroglobulinemia, and mantle cell lymphoma. Another 12 patients were diagnosed with monoclonal gammopathy of undetermined significance (MGUS). Convalescence levels of IgA, IgG2, or IgG4 were below the reference intervals in 16–20% of patients and 0–2% of controls. Three patients were diagnosed with a primary Ig deficiency, and seven patients started Ig replacement therapy. Our findings suggest that assessment of M protein and Ig levels could be considered in adults with IPD, as an episode of IPD may unmask a previously undiagnosed B-cell malignancy or immunodeficiency.

**Supplementary Information:**

The online version contains supplementary material available at 10.1038/s41598-026-61992-8.

## Introduction

Invasive pneumococcal disease (IPD) is a leading cause of morbidity and mortality globally^1^. By the time of diagnosis, the bacterium *Streptococcus pneumoniae* (the pneumococcus) has passed the host´s defense barriers into a sterile environment such as blood or cerebrospinal fluid. Bacterial survival is promoted by the surrounding polysaccharide capsule, described in at least 100 variants (serotypes), which confer resistance against phagocytosis and killing by phagocytes. Serotype-specific immunoglobulins (Ig), especially IgG subclass 2, protect against invading pneumococci^2^. By binding to capsule structures, the Ig opsonize the bacteria and activate the complement cascade, thereby facilitating phagocytosis and bacterial clearance^3^. Pneumococcal colonization or infection also induces production of Ig directed against bacterial surface proteins, contributing to protection against IPD^4^.

Primary and secondary Ig deficiencies are associated with increased risk of IPD. In most cases, an infection is the initial presentation of a primary immunodeficiency^5^. Secondary immunodeficiencies may develop as a consequence of any disease or treatment that affects the immune function, including hematological malignancies^6^. Patients with multiple myeloma are at particular risk of developing IPD^7^, primarily due to reduced levels and function of polyclonal Ig. This results from a monoclonal expansion of malignant plasma cells, producing large quantities of monoclonal and typically non-functional Ig (M protein), usually of IgG or IgA isotype. The clonal plasma cell expansion in the bone marrow suppresses the production of uninvolved polyclonal immunoglobulins, reduces antibody diversity, and leads to impaired humoral immunity. A broader immune dysregulation is also evident, involving many cell types, including T cells, NK cells and antigen-presenting cells^8,9^. Myeloma is commonly preceded by monoclonal gammopathy of undetermined significance (MGUS)^10^, defined by the presence of M protein in blood without fulfilling criteria for malignancy, although this condition is often not identified before the diagnosis of myeloma. However, there is evidence of progressive immune impairment from MGUS to multiple myeloma^8^, and both MGUS and myeloma patients have decreased levels of pneumococcal-specific Ig with reduced function^11^.

Although patients with impaired humoral immunity are known risk groups for IPD, less is known about the prevalence of hypogammaglobulinemia and monoclonal gammopathy among adult IPD patients. In this prospective multicenter study, we determined the presence of M protein and measured the concentrations of Ig and IgG subclasses and lymphocyte populations in Swedish adults with IPD, comparing them to age- and sex-matched controls.

## Results

### Patients and controls

**Fig. 1:**
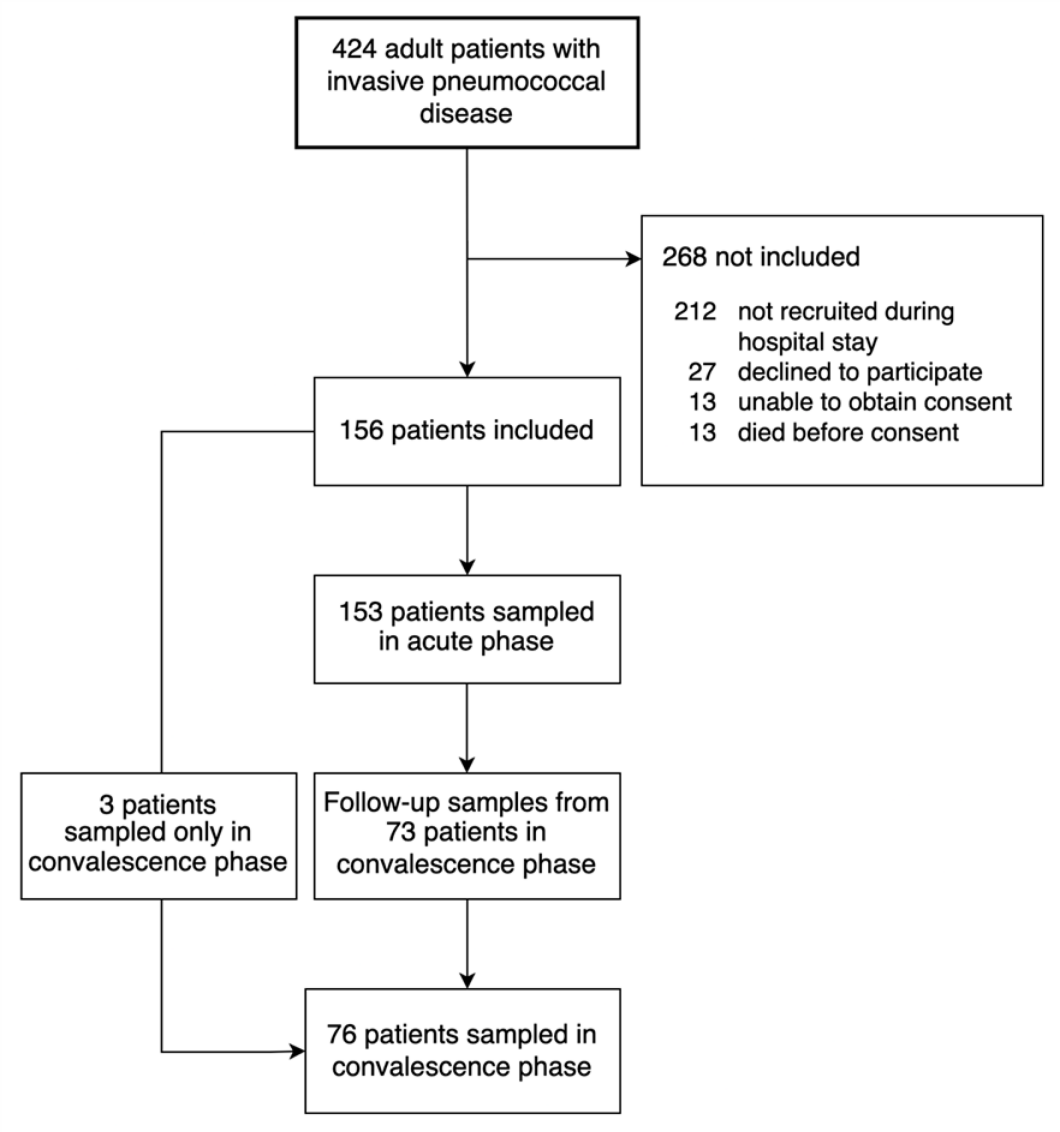
Flowchart of patient inclusion and analysis. Patients with invasive pneumococcal disease were sampled in the acute phase of infection, in the convalescent phase 2–4 months later, or at both time points.

Out of 424 eligible adult patients with IPD, 156 patients (37%) were included in the study (Fig. 1). Recruited patients had similar age and sex (median age 70 years, interquartile range [IQR] 59–77 years; 46% men) as non-included patients (median age 71 years, IQR 58–82 years; 48% men). Pneumonia was the most frequent IPD manifestation (84%), followed by meningitis (11%) (Table 1). At least one risk factor for IPD was found in 72% of the patients (Table 1). Twelve patients (8%) had a known hematological malignancy prior to inclusion. Criteria for septic shock were fulfilled in 11% of the patients, and the 30-day mortality was 3% (*n* = 4, all due to the pneumococcal infection) (Table 1).

**Table 1:** Clinical characteristics, infectious manifestations, and outcome in adult patients with invasive pneumococcal disease (IPD) sampled either during the acute phase of infection, in the convalescent phase 2–4 months later, or at both time points, and controls.

Characteristic	Patients									Controls
	Acute phase (*n* = 153)	Acute phase only (*n* = 80)^a^		Convalescent phase (*n* = 76)^a^			All (*n* = 156)			(*n* = 64)
Sex, *n* (%)										
Men	72 (47)	36 (45)		36 (47)			72 (46)			28 (44)
Age in years, median (range)	70 (18–92)	70 (29–92)		72 (18–89)			70 (18–92)			68 (23–97)
Age groups, *n* (%)										
18–49 years	19 (12)	9 (11)		10 (13)			19 (12)			8 (12)
50–64 years	35 (23)	20 (25)		16 (21)			36 (23)			14 (22)
65–79 years	69 (45)	34 (43)		37 (49)			71 (45)			32 (50)
≥ 80 years	30 (20)	17 (21)		13 (17)			30 (19)			10 (16)
Vaccinated with a pneumococcal vaccine^b^	32 (21)	12 (15)		20 (26)			32 (21)			9 (16)
Risk factors, *n* (%)^c^										
Presence of ≥ 1 risk factor^d^	111 (73)	63 (79)		49 (65)			112 (72)			11 (17)
Number of risk factors (median, range)	2 (1–6)	2 (1–5)		2 (1–6)			2 (1–6)			1 (1)
Cardiovascular disease	49 (32)	28 (35)		21 (28)			49 (31)			7 (11)
Pulmonary disease	35 (23)	23 (29)		13 (17)			36 (23)			2 (3)
Smoking^e^	48 (31)	34 (43)		14 (18)			48 (31)			0 (0)
Diabetes mellitus	27 (18)	14 (18)		13 (17)			27 (17)			1 (2)
Malignancy	22 (14)	12 (15)		10 (13)			22 (14)			0 (0)
Solid tumor	10 (7)	5 (6)		5 (7)			10 (6)			0 (0)
With ongoing immunosuppressive therapy	3 (2)	1 (1)		2 (3)			3 (2)			0 (0)
Hematological malignancy	12 (8)	7 (9)		5 (7)			12 (8)			0 (0)
With ongoing immunosuppressive therapy	4 (3)	2 (3)		2 (3)			4 (3)			0 (0)
Immune deficiency^f^	2 (1)	1 (1)		1 (1)			2 (1)			0 (0)
Immunosuppressive treatment^g^ (excluding those with malignancy as shown above)	6 (4)	4 (5)		2 (3)			6 (4)			0 (0)
Alcohol or substance abuse, or both	9 (6)	7 (9)		2 (3)			9 (6)			0 (0)
Renal disease	9 (6)	5 (6)		4 (5)			9 (6)			0 (0)
Autoimmune disease	8 (5)	5 (6)		3 (4)			8 (5)			1 (2)
Liver disease	4 (3)	3 (4)		1 (1)			4 (3)			0 (0)
Asplenia	3 (2)	2 (3)		1 (1)			3 (2)			0 (0)
Pregnancy	0 (0)	0 (0)		0 (0)			0 (0)			0 (0)
Other^h^	4 (3)	2 (3)		2 (3)			4 (3)			0 (0)
Clinical manifestation during the IPD episode, *n* (%)										
Pneumonia	129 (84)	67 (83)		64 (84)			131 (84)			NA
Meningitis	16 (11)	10 (13)		7 (9)			17 (11)			NA
Bacteremia without focus	7 (5)	3 (4)		4 (5)			7 (5)			NA
Other^i^	14 (9)	8 (10)		6 (8)			14 (9)			NA
Clinical course and outcome, *n* (%)										
Sepsis^j^	106 (69)	57 (71)		50 (66)			107 (69)			NA
Septic shock^j^	17 (11)	14 (18)		3 (4)			17 (11)			NA
Admittance to intensive care unit	36 (24)	25 (31)		12 (16)			37 (24)			NA
Mechanical ventilation	18 (12)	13 (16)		6 (8)			19 (12)			NA
Non-invasive mechanical ventilation	27 (18)	18 (23)		10 (13)			28 (18)			NA
Complications	61 (40)	43 (54)		19 (25)			62 (40)			NA
Sequelae	11 (7)	8 (10)		3 (4)			11 (7)			NA
30-day mortality^k^	4 (3)	4 (5)		NA			4 (3)			NA
1-year mortality^k^	20 (13)	15 (19)		5 (7)			20 (13)			NA

NA, not applicable.

^a^ Seventy-three patients provided samples during both the acute and convalescent phases. Three patients were sampled only during the convalescent phase, whereas 80 were sampled only during the acute phase and did not provide a follow-up sample.

^b^ Vaccinated with a pneumococcal conjugate, polysaccharide vaccine, or both. Missing data for six control individuals.

^c^ IPD risk factors at study inclusion.

^d^ Any of the IPD risk factors listed below.

^e^ Active smoking or smoking cessation within ten years before the IPD episode.

^f^ Allogeneic stem cell transplantation (*n* = 1), autologous stem cell transplantation (*n* = 1).

^g^ Glucocorticoids > 5 mg/day for more than two weeks (*n* = 5), myeloma treatment (*n* = 4) such as bendamustine, bortezomib/dexamethasone, methotrexate (*n* = 3), mycophenolate mofetil (*n* = 1), rituximab (*n* = 1), and etanercept (*n* = 1).

^h^ Surgery for meningioma (*n* = 2), ventricular-peritoneal shunt (*n* = 1), surgery for cholesteatoma (*n* = 1).

^i^ Acute media otitis (*n* = 6), septic arthritis (*n* = 4), epidural abscess (*n* = 1), epidural abscess and spondylodiscitis (*n* = 1), mastitis (*n* = 1), distal shunt infection (*n* = 1).

^j^ As defined by the third international consensus definitions for sepsis and septic shock (sepsis-3, 2016).

^k^ Death within 30 days or one year from sampling a sterile site in which *Streptococcus pneumoniae* was identified by culture or PCR.

Blood was drawn from 153 patients in the acute phase of the infection, of whom 73 were also sampled during convalescence 2–4 months later (Fig. 1) at a median of 84 days after the IPD diagnosis (IQR 65–105 days). Three patients donated blood only in the convalescence phase. Out of 65 recruited age and sex-matched controls, one individual was excluded because of ongoing immunosuppressive treatment (prednisolone 20 mg daily) at the time of sampling, resulting in 64 evaluated controls (Table 1). At study inclusion, none of the patients or controls were receiving Ig replacement therapy.

### Monoclonal gammopathy and B-cell malignancy

**Fig. 2:**
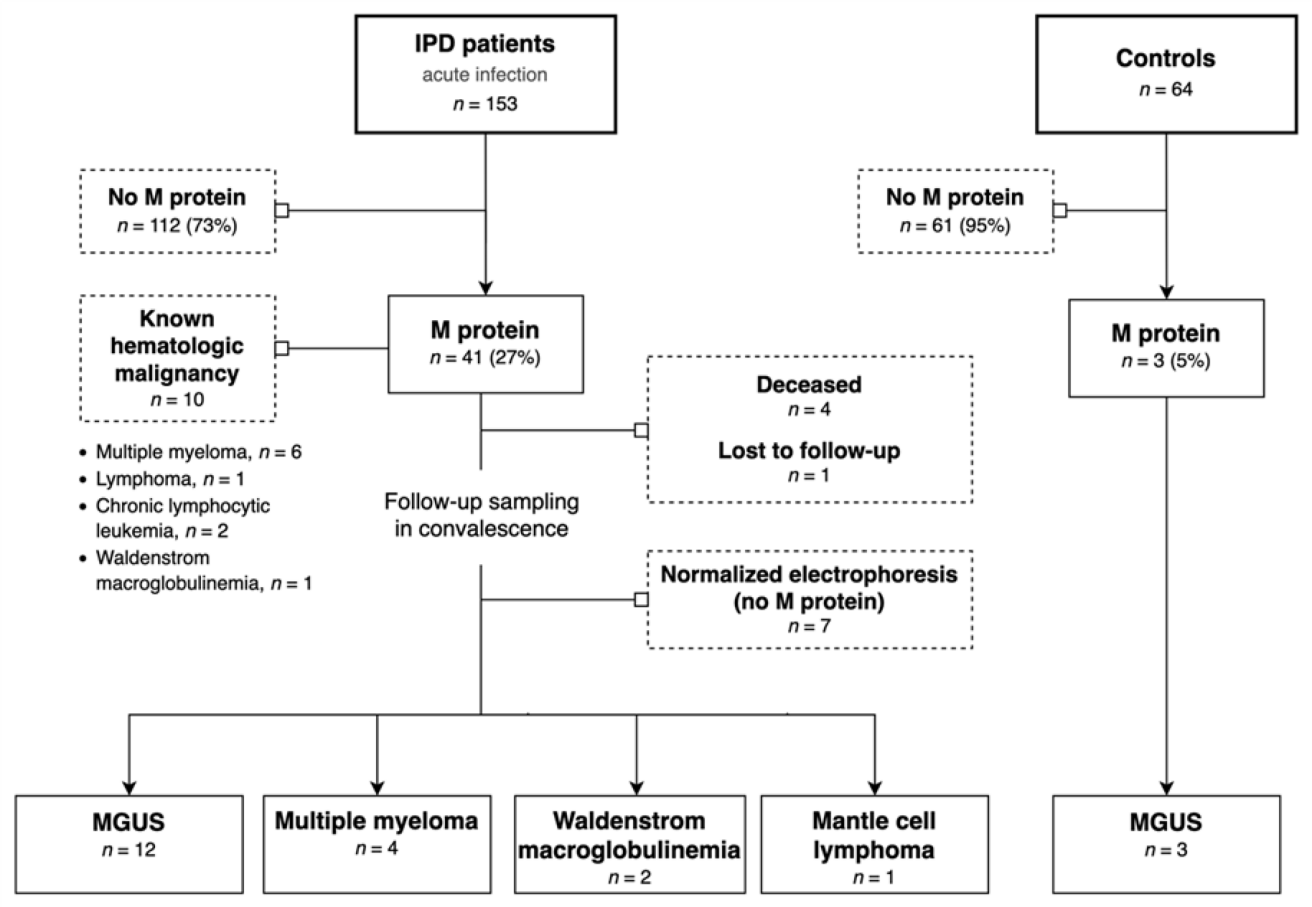
Flowchart presenting results from protein electrophoresis detecting M protein in serum from patients with acute infection and control individuals, respectively, and outcome (B-cell malignancy or MGUS) after follow-up analysis. IPD, invasive pneumococcal disease; M protein, monoclonal protein; MGUS, monoclonal gammopathy of uncertain significance.

Monoclonal immunoglobulins (M protein) were detected in 27% of patients (41/153) during acute infection and in 4.7% of controls (3/64; *p* < 0.001) (Fig. 2). The majority of patients with M protein were men (66%; *p* = 0.03). Other factors, including age, risk factors for IPD, manifestations, and outcomes, were similarly distributed between patients with and without M protein (Supplementary Table S1). After adjustment for age, sex, and predisposing conditions, IPD patients had eightfold higher odds of M protein detection than non-hospitalised controls (aOR 7.96, 95% CI 2.22–28.52, *p* = 0.001).

In ten patients with detected M protein, a hematological malignancy was known at inclusion (Fig. 2 and Supplementary Table S2). Hence, M protein was detected in 22% (31/141; *p* = 0.002) of patients without previously known hematological malignancy. In seven of these patients, the M protein was transient and no longer detectable during convalescence (Fig. 2). Another four patients with M protein in the acute phase died, and one was lost before follow-up. Among the remaining patients with persistent M protein (*n* = 19) a hematological malignancy was diagnosed in seven patients (multiple myeloma, *n* = 4; Waldenstrom macroglobulinemia, *n* = 2; mantle cell lymphoma, *n* = 1) and MGUS in 12 patients (Fig. 2). Thus, among included patients without previously known hematological malignancy, 4.9% (7/144) were diagnosed with a B-cell malignancy, and another 8.3% (12/144) with MGUS. M protein levels in patients with MGUS ranged from 0.5 to 11 g/L (median 3 g/L), and from 2 to 40 g/L (median 10 g/L) in patients with a newly diagnosed B-cell malignancy (Supplementary Table S2). The median age of the patients with MGUS and newly discovered B-cell malignancy was 76 and 76 years, respectively, the majority being men presenting with pneumonia (Supplementary Table S2). Five patients with newly diagnosed B-cell malignancy met sepsis criteria, and three were admitted to the ICU, one of whom had septic shock and required mechanical ventilation. The median length of hospital stay was 5 days (range 1–27) in the MGUS group and 6 days (range 4–106) among the patients with newly diagnosed B-cell malignancy. One patient with a new diagnosis of B-cell malignancy died within one year of the IPD episode. Furthermore, one patient with an IgG kappa M protein of 12 g/L died within 30 days of inclusion and therefore did not undergo further evaluation for MGUS or a B-cell malignancy. None of the 12 patients with a newly diagnosed MGUS died during follow-up.

### Ig levels

**Fig. 3:**
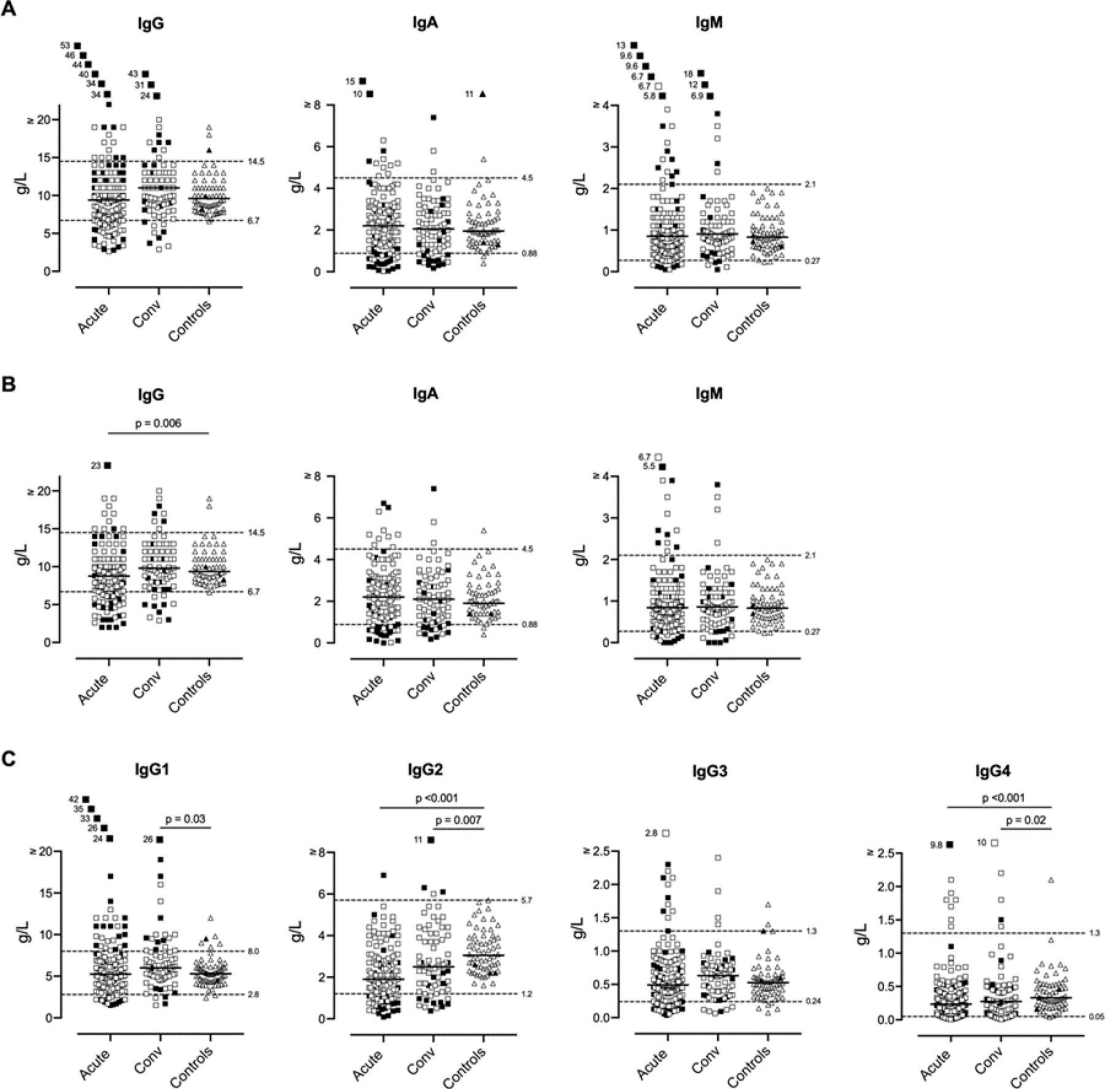
Concentrations of serum immunoglobulin (Ig)G, IgA, IgM, and IgG subclasses 1–4 in invasive pneumococcal disease patients during acute infection, in the convalescent phase 2–4 months later (Conv), and in controls. (**A**) IgG, IgA, and IgM levels including monoclonal Ig (M protein), if any. Acute, *n* = 153; Conv, *n* = 76; Controls, *n* = 64. (**B**) Adjusted IgG, IgA, and IgM levels with exclusion of the M protein fraction. Acute, *n* = 146; Conv; *n* = 75; Controls, *n* = 64. (**C**) IgG subclasses 1–4 including M protein, if any. Acute, *n* = 152; Conv; *n* = 75; Controls, *n* = 64. (**A**–**C**) Each patient is represented by a square, each control person by a triangle. Individuals with detected M protein are marked with a black symbol. The horizontal black lines represent the median values. Dashed horizontal lines indicate the upper and lower limits of the reference interval of each Ig and IgG subclass, respectively.

The total levels of IgG, IgA, and IgM, including M protein, if any, in patients sampled in the acute phase of infection, in convalescence 2–4 months after the IPD episode, or at both occasions, and in controls are shown in Fig. 3A. Adjusted Ig levels excluding any M protein fraction are shown in Fig. 3B. Except for slightly lower IgG levels in the acute phase of the infection, there were no significant differences in IgG, IgA or IgM concentrations between patients and controls (Fig. 3A and B). In contrast, IgG2 levels were significantly lower in patients than controls during both the acute and convalescent phases (Fig. 3C). Minor but statistically significant differences in IgG4 levels were also observed when comparing IPD patients in the acute and convalescent phases with controls (Fig. 3C).

Almost half of the patients (44%) sampled in the acute phase and 34% in convalescence had levels of at least one Ig or IgG subclass below the lower limit of the reference interval, compared to 16% of the controls (values unadjusted for M protein). IgG, IgG2, IgG4 or IgA levels below the reference interval were observed in 16–21% of patients during acute infection and 12–20% in the convalescence phase (Table 2). The number of patients with low IgG levels was even higher after exclusion of the M protein fraction from the Ig concentrations (Table 2). Concomitant low levels of two or more Ig isotypes were most frequently observed for IgG and IgA (Supplementary Figure S1).

**Table 2:** The number and proportion of patients with invasive pneumococcal disease (IPD) and controls with immunoglobulin (Ig) levels below the lower limit of the reference interval of each Ig isotype and IgG subclass, respectively.

Immunoglobulin	Below lower limit of reference interval^a^, *n* (%)		
	Acute	Convalescence	Controls
*Concentrations of IgG*,* IgA*,* IgM including M protein fraction*,* where applicable*^*b*^			
IgG	31 (20) ***	9 (12) *	1 (2)
IgA	25 (16) **	15 (20) **	2 (2)
IgM	14 (9)	5 (7)	2 (2)
*Concentrations of IgA*,* IgM*,* IgG without M protein fraction*^*c*^			
IgG	38 (26) ***	10 (13) *	1 (2)
IgA	22 (15) *	13 (17) *	2 (3)
IgM	10 (7)	4 (5)	2 (3)
*IgG subclasses including M protein fraction*,* where applicable*^*d*^			
IgG1	17 (11)	4 (5)	2 (2)
IgG2	32 (21) ***	12 (16) ***	0 (0)
IgG3	28 (18)	6 (8)	5 (8)
IgG4	25 (16) **	12 (16) **	1 (2)

^a^ Reference intervals: IgG 6.7–14.5 g/L; IgA 0.88–4.5 g/L; IgM 0.27–2.1 g/L; IgG1 2.8–8.0 g/L; IgG2 1.2–5.7 g/L; IgG3 0.24–1.3 g/L; IgG4 0.05–1.3 g/L.

^b^ Acute phase *n* = 153; convalescent phase 2–4 months later *n* = 76; controls *n* = 64.

^c^ Acute phase *n* = 146; convalescent phase *n* = 75; controls *n* = 64.

^d^ Acute phase *n* = 153; convalescent phase *n* = 76; controls *n* = 64.

* *p* < 0.05; ** *p* < 0.01; *** *p* < 0.001; patients vs. controls. All comparisons were performed using Fisher’s exact test. Significant values are in bold.

Five patients with MGUS and six patients with newly diagnosed hematological malignancies had hypogammaglobulinemia with at least one non-monoclonal Ig isotype below the reference interval (Supplementary Table S2).

Paired data, not adjusted for M protein, from 73 patients analyzed during acute infection and convalescence showed significantly higher levels of IgG and IgG subclasses 1–4 in convalescence compared to the acute phase. In contrast, IgA and IgM levels remained unchanged (Supplementary Figure S2).

### Immunodeficiencies and Ig replacement therapy

Follow-up investigation of patients with Ig or Ig subclass levels below the lower limit of the reference interval resulted in a diagnosis of primary immunodeficiency in two patients due to low levels of Ig (deficiency of IgG2 and IgG3 in one patient and IgG and IgA in the other) and a history of repeated bacterial respiratory tract infections. Both were started on Ig replacement therapy, as was another patient diagnosed with primary immunodeficiency in childhood. One patient was diagnosed with selective IgA deficiency, and four additional patients were diagnosed with secondary immunodeficiency following known hematological malignancy or previous Rituximab treatment and started Ig replacement therapy.

### Blood lymphocyte concentrations

**Fig. 4:**
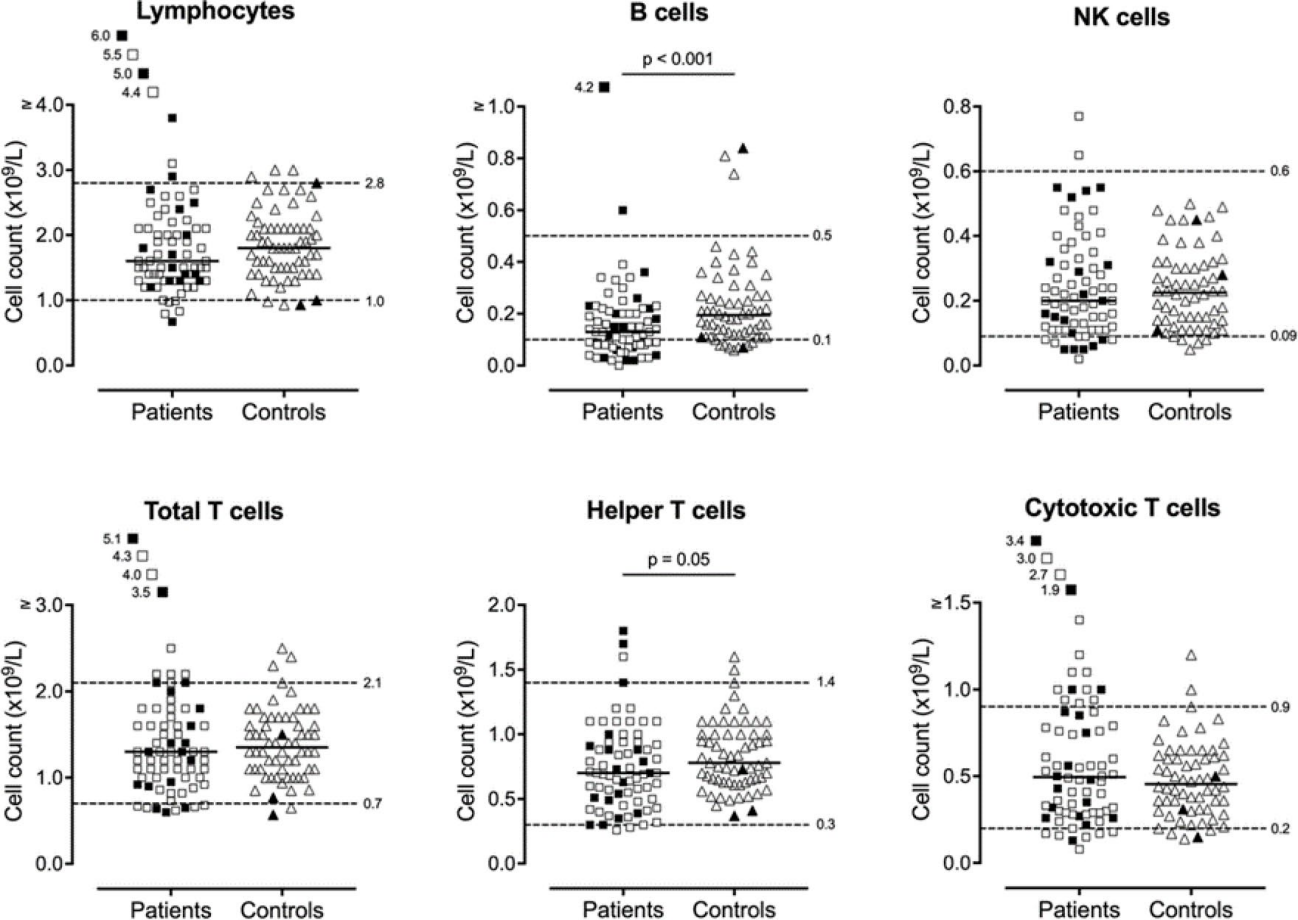
Blood cell count (x10^9^/L) of total lymphocytes, B cells (CD19+), NK cells (CD3-CD19-CD56+CD16+ or CD3-CD19-CD56+CD16-), T cells (CD3+), helper T cells (CD3+CD4+), and cytotoxic T cells (CD3+CD8+) in patients sampled in the convalescent phase 2–4 months after the invasive pneumococcal disease episode (*n* = 70) and controls (*n* = 62). Each individual is represented by one symbol and the horizontal lines represent the median values. Individuals with M protein are marked as black squares (patients) or black triangles (controls). Dashed horizontal lines indicate the upper and lower limits of the reference interval.

B cell concentrations in blood, measured in the convalescent phase of IPD, were significantly lower in patients as compared with controls (*p* < 0.001) (Fig. 4), and 40% of patients, compared to 13% of controls, had concentrations below the lower limit of the reference interval (28/70 vs. 8/62; *p* < 0.001). While patients also tended to have slightly lower T helper cell counts than controls, no significant differences were observed for total lymphocytes, total T cells, cytotoxic T cells, nor NK cells between patients and controls (Fig. 4). Nor were there any correlations between Ig levels and concentrations of B or T cells (Supplementary Table S3), or any association between the presence of M protein and lymphocyte concentrations (Fig. 4).

## Discussion

To our knowledge, this is the largest published prospective study evaluating immune deficiencies in adults with IPD. Among 156 IPD patients, seven (4.5%) were identified with previously unknown B-cell malignancy, 12 (7.7%) with MGUS, and eight patients with primary or secondary immunodeficiency (5.1%), of whom seven required Ig replacement therapy. Due to lack of routine assessments of M protein, Ig, or IgG subclass levels in the standard care of patients with a single IPD episode, these patients are rarely identified in this context today.

Patients with hematological malignancies, especially multiple myeloma, have a very high risk of IPD^7,12^. Previous case reports indicate that IPD might be the first sign of an undiagnosed hematological malignancy, supported by a French study^13,14^. Epidemiological studies further show that IPD patients have an increased risk of developing myeloma within a few years following the infection, compared to non-IPD individuals^15,16^.

The treatment landscape for hematological malignancies has expanded significantly over the past two decades. In the case of multiple myeloma, proteasome inhibitors, immunomodulatory drugs, and monoclonal antibodies targeting plasma cells have been incorporated into the standard of care^17^. More recently, bispecific antibodies and Chimeric Antigen Receptor (CAR)-T cells targeting B-cell maturation antigen have been introduced as therapeutic options^18,19^. These advancements have significantly improved the survival rates of myeloma patients^20^, and early discovery is crucial for optimizing the care and prognosis of these patients.

This study found MGUS in 8.3% of IPD patients without previously known hematological malignancy as compared with 4.7% among the controls and an estimated 4.4% in the general population above 50 years, as recently demonstrated in a nationwide Icelandic study^21^. Because patients with MGUS are typically asymptomatic, the condition often remains undiagnosed. However, its detection may be advantageous for the individual due to the increased risk of infection associated with MGUS as well as the small but not insignificant risk of malignant transformation (up to 1% annually)^22^. Data indicate that multiple myeloma patients with a prior MGUS diagnosis have increased survival compared to those without previously known MGUS^23^, likely due to close monitoring and early treatment. Immune dysregulation is present already at the MGUS stage. Uninvolved polyclonal immunoglobulins may be suppressed, and early impairments in both innate and cellular immunity have been described^8,9^. Although these abnormalities are less pronounced than in multiple myeloma, they may still be clinically relevant. Accordingly, pneumococcal vaccination is recommended for patients with MGUS^24^. However, high M protein concentrations are associated with inferior vaccine responses^25^, underscoring the importance of early MGUS diagnosis, enabling pneumococcal vaccination at an early stage.

M protein was detected in 27% of all included patients during acute infection, and in 22% of those without previously known hematological malignancy. These are higher frequencies than previously observed (7–9%) in patients with invasive disease caused primarily or exclusively by pneumococci^13,26^. In seven cases, M protein was undetectable at follow-up 2–4 months after the acute infection. Transient M proteins have previously been described during infections caused by other microbes^27^, but also in autoimmune or malignant disorders as well as obstructive pulmonary conditions^28^.

The ability to detect low levels of M protein may vary between analytical methods, with a potentially increased likelihood of detecting low-abundance monoclonal proteins by the use of high-sensitivity mass spectrometry^29^. The clinical significance of such findings, particularly regarding susceptibility to infections and the risk of malignant transformation, remains to be determined in larger prospective studies.

In this study, low levels of IgG were observed in 26% of the patients during acute infection and 13% in convalescence, comparable to prior studies^13,26,30^. In the paired Ig analysis, both IgG and all IgG subclass levels were lower in the acute than the convalescence phase. Low IgG concentrations during acute infection have previously been observed in patients with IPD^26^ and septic shock^31^. Suggested mechanisms include Ig consumption through antigen-antibody binding or capillary leakage. Thus, low Ig levels in the acute phase of IPD should be confirmed upon recovery. Significantly lower levels of IgG2 and IgG4 were found in patients as compared to controls, both during acute infection and convalescence. IgG2 constitutes a major fraction of the antibodies targeting encapsulated bacteria, including pneumococci^32^. The importance of IgG4 is less understood. However, both IgG2 and IgG4 are induced by polysaccharides^3^, and IgG4 deficiency may be associated with recurrent respiratory infections^33^. Though being the most common Ig deficiency in the general population, only one patient was diagnosed with primary IgA deficiency.

Four study patients (3%) had a primary immunodeficiency, including one diagnosed prior to the study. This prevalence is slightly lower than the previously reported 4–8%^13,26^, and considerably lower than in a retrospective study (17%) assessing younger adults with invasive infections caused by encapsulated bacteria in France^34^. The variation in prevalence may partly be explained by differences in the diagnostic criteria. For example, the European Society for Immunodeficiencies (ESID) guidelines applied in this study require repeated Ig measurements during infection-free intervals^35^.

We found significantly lower levels of circulating B cells in IPD patients during convalescence compared to controls. There was no association between concentrations of B cells and Ig, illustrating that Ig levels do not simply reflect circulating B cell numbers. However, our analysis was limited to total CD19 + B cells and did not include clonal plasma cells, which may be increased in patients with myeloma or MGUS^25,36^ and are associated with disease progression and poorer survival^36^. A trend toward reduced T helper cell concentrations was also observed. The modest reductions in B cells and T helper cells observed 2–4 months after IPD may reflect a sustained post-infectious effect on the adaptive immune system with incomplete recovery. Lymphopenia is commonly observed in hospitalized patients with acute pneumococcal pneumonia^37^, and sepsis is known to induce lymphocyte apoptosis and altered lymphocyte tissue distribution^38^. B cell depletion has been shown to persist for at least 28 days following septic shock^39^, although the longer-term effects of invasive infections on B cell counts remain poorly characterized. In our cohort, some patients with M protein had reduced peripheral B cells, although this was not observed consistently across all cases. Additional factors, including comorbidities and treatments affecting B cell homeostasis, may have contributed to the observed differences between patients and controls, but this could not be assessed from our data.

Strengths of this study include the relatively large study size and the prospective, multicenter design with age- and sex-matched controls. Extensive clinical data were collected from each included patient. The presence of M protein, Ig levels, and lymphocyte populations were analyzed at the same accredited laboratory for all patients and controls. Furthermore, both acute and convalescent blood samples were collected, with the latter obtained within a uniform follow-up interval.

Several limitations warrant consideration. Only 37% of eligible patients were enrolled in the study. Furthermore, only half of the enrolled patients provided follow-up samples, potentially introducing further selection bias towards healthier individuals in the convalescence phase. However, the demographic data of enrolled patients, including comorbidities and age distribution, were comparable to previously reported data from all IPD patients in the same region^7^. The one-month case fatality rate of 3% was lower in the study population compared to previous data from the same area (13%)^40^, and patients with the highest morbidity may thus be underrepresented. Serum free light chains as well as the presence of Bence-Jones proteinuria were not assessed in this study, which may have led to underdetection of light-chain MGUS. Although the controls were matched by age and sex, they were not recruited from a hospitalized population and had fewer underlying comorbidities than the patient group; therefore, comparisons should be interpreted with caution.

## Conclusions

Previously unknown M protein and hypogammaglobulinemia are prevalent among patients with IPD, and an episode of IPD may be the first sign of malignant B-cell disease or a pre-malignant B-cell condition. Our findings suggest that assessment of M protein and Ig could be considered in adult patients following an episode of IPD. Further studies in larger, well-characterized cohorts, ideally incorporating more comprehensive and sensitive analytical approaches, are needed to confirm these findings and clarify which patient subgroups may benefit most from such investigations.

## Materials and methods

### Study participants

Adult IPD patients were prospectively recruited between March 2018 and September 2023 at NU Hospital Group, Trollhättan, and Södra Älvsborg Hospital, Borås, and between January 2022 and April 2023 at Sahlgrenska University Hospital (SU), Gothenburg, together serving 1,290,000 residents in the Region Västra Götaland, Sweden. Inclusion criteria were age ≥ 18 years, and detection of pneumococci by culture in blood or cerebrospinal fluid (CSF), or by PCR in CSF, at any of the clinical laboratories at the participating hospitals. Molecular CSF analyses were performed using the BioFire FilmArray Meningitis/Encephalitis Panel on the BioFire FilmArray Torch System (both from Biomérieux, Marcy-l’Étoile, France)^41^, or by 16 S rRNA gene detection with subsequent sequencing^42^. A study physician or nurse was notified by the laboratory of new IPD cases, and written informed consent was obtained from patients, or in case of severe disease, from a first-line relative, before inclusion. The study was approved by the Regional Ethics Committee of Gothenburg (894−17, T616–18) and the Swedish Ethical Review Authority (2020–06391).

The patients were interviewed about pneumococcal vaccination and smoking within ten years, and the Alcohol Use Disorders Identification Test (AUDIT) was performed. Data collected from medical records included pneumococcal vaccination, risk factors for IPD, clinical manifestations including sepsis or septic shock, and outcomes. Case fatality rate was defined as death within 30 days and 1 year, respectively, from the date of sampling with pneumococcal detection.

Non-hospitalized age- and sex-matched individuals were enrolled as controls after providing written informed consent. AUDIT was performed, and information about health status, current medication, and chronic diseases was obtained through interviews. Exclusion criteria for controls were fever within the past two weeks, ongoing immunosuppressive treatment or disease, or a history of IPD.

### Ig and M protein analysis

Blood was drawn from patients in the acute phase and in convalescence 2–4 months later. Control individuals were sampled at outpatient clinics. Levels of IgG, IgA, IgM, and IgG subclasses were quantified at the Department of Clinical Immunology and Transfusion Medicine, SU, using turbidimetry (Optilite, Binding Site, Birmingham, UK) according to the manufacturer´s instructions. Reference intervals are presented in Supplementary Table S4. Presence of M protein was detected by serum protein gel electrophoresis (Sebia Hydragel 15 h by Sebia, Lisses, France), and further typed by immunofixation using antibodies against IgG, IgA, IgM, kappa, and lambda (Sebia). M protein was quantified by protein capillary electrophoresis (Capillarys 3 Tera, Sebia) at the Department of Clinical Chemistry, SU. To determine polyclonal IgG levels in patients with M protein of IgG isotype, the M protein concentration was subtracted from total IgG levels. Adjusted levels of Ig subclasses could not be determined. In cases with M protein of IgA or IgM isotypes, polyclonal Ig levels could not be distinguished and were assigned a concentration of 0 g/L. The volume of seven samples with detected M protein was too small to allow quantification.

Selective IgA deficiency was defined as IgA < 0.07 g/L, with normal or high levels of IgG and IgM. Diagnosis of IgG subclass deficiency required low levels of at least one IgG subclass, measured twice during infection-free periods, and a history of repeated bacterial infections, as defined by the European Society for Immunodeficiencies (ESID)^35^.

### Lymphocyte analysis

Lymphocyte populations (CD3+ T cells, CD3+CD4+ helper T cells, CD3+CD8+ cytotoxic T cells, CD19+ B cells, and CD3-CD19-CD56+CD16+ or CD3-CD19-CD56+CD16- NK cells) were analyzed by flow cytometry in 50 µL of EDTA blood obtained from patients in the convalescent phase 2–4 months after acute infection and from controls. Samples were analyzed at the immunology laboratory within 24 h of collection using BD FACSCanto or BD FACSLyric flow cytometers (BD Biosciences, Franklin Lakes, NJ) and BD multitest 6-color TBNK reagent with Trucount tubes (BD Biosciences) according to the manufacturer´s instructions. Reference intervals are listed in Supplementary Table S5.

### Statistical analysis

Differences between groups were analyzed using the Mann–Whitney U test, paired data using the Wilcoxon signed-rank test, and categorical data using Fisher’s exact or Pearson’s chi-square test. Correlations between Ig levels and lymphocyte population counts were assessed using Spearman’s rank correlation test. The association between IPD and M protein detection was assessed using multivariable logistic regression analysis adjusted for age, sex, and predisposing conditions. The result was reported as adjusted odds ratio (aOR) with 95% confidence interval (CI). All calculations were two-sided with alpha 0.05 performed in GraphPad Prism version 10.2.1 (GraphPad Software, Boston, MA) and IBM SPSS Statistics version 30 (IBM Corporation, Armonk, NY).

## Supplementary Information

Below is the link to the electronic supplementary material.

Supplementary Material 1

## Data Availability

The anonymized dataset used in this study is available from the corresponding author upon request.

## Abbreviations

AUDIT: Alcohol use disorders identification test
CI: Confidence interval
CSF: Cerebrospinal fluid
ESID: European Society for Immunodeficiencies
ICU: Intensive care unit
Ig: Immunoglobulin
IPD: Invasive pneumococcal disease
IQR: Interquartile range
M protein: Monoclonal protein
MGUS: Monoclonal gammopathy of undetermined significance
OR: Odds ratio
PCR: Polymerase chain reaction
SU: Sahlgrenska University Hospital
