# Supplementary material for: Invasive pneumococcal disease unmasks monoclonal immunoglobulins and antibody deficiencies: a multicenter prospective study in adults
Source: Sci Rep. 2026 Jul 24;16:23203. doi: 10.1038/s41598-026-61992-8 (PMC13400745; doi:10.1038/s41598-026-61992-8)
Supplement: Supplementary file 1 — Supplementary Material 1 [file 41598_2026_61992_MOESM1_ESM.docx]

# Supplementary Material

**Invasive pneumococcal disease unmasks monoclonal immunoglobulins and antibody deficiencies: a multicenter prospective study in adults**

Tor Härnqvist^1,2,*^, Karin Bergman^1,3,*^, Åsa Mellgren^1,4^, Magnus Brink^1,4^, Amanda Nilsson^2^, Staffan Nilsson^5^, Bengt Andersson^6^, Rune Andersson^1,7^, Anna Lundgren^6,8^, Johanna Karlsson^1,2^, Susann Skovbjerg^1,7^

**Supplementary Table S1.** Clinical characteristics, infectious manifestations and outcome of the IPD episode in patients with or without detected monoclonal immunoglobulins (M protein) in serum.

| Characteristic | M protein (*n*=41) | No M protein (*n*=115)^a^ | *p*-value |
| --- | --- | --- | --- |
| Sex, *n (*%) | | | |
| Men | 27 (66) | 45 (39) | **0.03** |
| Age in years, median (range) | 72 (33–89) | 69 (18–92) | NS |
| Age groups, *n* (%) | | | |
| 18–49 years | 3 (7) | 16 (14) | NS |
| 50–64 years | 7 (17) | 29 (25) | NS |
| 65–79 years | 22 (54) | 49 (43) | NS |
| ≥80 years | 9 (22) | 21 (18) | NS |
| Vaccinated with a pneumococcal vaccine^b^ | 9 (22) | 23 (20) | NS |
| Risk factors, *n (*%)^c^ | | | |
| Any risk factor^d^ | 30 (73) | 82 (71) | NS |
| Number of risk factors (median, range) | 2 (1–6) | 2 (1–5) | NS |
| Cardiovascular disease | 16 (39) | 33 (29) | NS |
| Pulmonary disease | 9 (22) | 27 (24) | NS |
| Smoking^e^ | 9 (22) | 39 (34) | NS |
| Malignancy | 14 (34) | 8 (7) | NS |
| Solid tumor | 4 (10) | 6 (5) | NS |
| Hematological | 10 (24) | 2 (2) | **<.001** |
| Diabetes mellitus | 5 (12) | 22 (19) | NS |
| Immune deficiency^f^ | 1 (2) | 1 (1) | NS |
| Immunosuppressive treatment^g^ | 4 (10) | 9 (8) | NS |
| Alcohol and/or substance abuse | 9 (6) | 2 (3) | NS |
| Renal disease | 4 (10) | 5 (4) | NS |
| Autoimmune disease | 0 (0) | 8 (7) | NS |
| Liver disease | 0 (0) | 4 (4) | NS |
| Asplenia | 0 (0) | 3 (3) | NS |
| Other^h^ | 0 (0) | 4 (4) | NS |
| Clinical manifestation, *n* (%) | | | |
| Pneumonia | 36 (88) | 95 (83) | NS |
| Meningitis | 2 (5) | 15 (13) | NS |
| Bacteremia without focus | 3(7) | 4 (4) | NS |
| Other^i^ | 5 (12) | 9 (8) | NS |
| Clinical course and outcome, *n* (%) | | | |
| Sepsis^j^ | 30 (73) | 94 (82) | NS |
| Septic shock^j^ | 6 (15) | 11 (9.6) | NS |
| Admittance to intensive care unit | 12 (29) | 25 (22) | NS |
| Mechanical ventilation | 4 (10) | 15 (13) | NS |
| Non-invasive mechanical ventilation | 10 (24) | 18 (16) | NS |
| Complications | 18 (44) | 44 (38) | NS |
| Sequelae | 1 (2) | 10 (9) | NS |
| 30-day mortality^k^ | 2 (5) | 2 (2) | NS |
| 1-year mortality^k^ | 8 (20) | 12 (10) | NS |

IPD invasive pneumococcal disease; NS, not statistically significant

^a^ One hundred and twelve patients with no detected M protein were sampled in the acute phase and three patients were exclusively sampled in the convalescent phase.

^b^ Vaccinated with a pneumococcal conjugate or polysaccharide vaccine, or both.

^c^ IPD risk factors before study inclusion.

^d^ Any of the IPD risk factors listed below.

^e^ Active smoking or smoking cessation within ten years before the IPD episode.

^f^ Allogeneic stem cell transplantation (*n*=1), autologous stem cell transplantation (*n*=1).

^g^ Glucocorticoids >5 mg/day for more than two weeks or other immunosuppressants.

^h^ Surgery for meningioma (*n*= 2), ventricular-peritoneal shunt (*n*= 1), surgery for cholesteatoma (*n*= 1).

^i^ Acute media otitis (*n*=6), septic arthritis (*n*=4), epidural abscess (*n*=1), epidural abscess and spondylodiscitis (*n*=1), mastitis (*n*=1), distal shunt infection (*n*=1).

^j^ As defined by The Third International Consensus Definitions for Sepsis and Septic Shock (Sepsis-3, 2016).

^k^ Death within 30 days or 1 year from sampling a sterile site in which *Streptococcus pneumoniae* was identified by culture or PCR.

**Supplementary Table S2.** Characteristics of patients with detected M protein and previously known hematological malignancy, newly discovered B-cell malignancy and newly diagnosed monoclonal gammopathy of undetermined significance (MGUS), the latter two following detection of M protein in the study.

|  |  |  |  |  |  | | Serum immunoglobulin levels^a^, g/L | | | | | | | | | | |
| --- | --- | --- | --- | --- | --- | --- | --- | --- | --- | --- | --- | --- | --- | --- | --- | --- | --- |
| Sex | Age interval (years) | IPD manifestation | Monoclonal protein, Ig type (concentration) | Diagnosis, outcome | Sample type | | IgG | | IgA | | IgM | IgG1 | | IgG2 | IgG3 | | IgG4 |
| B-cell malignancy known at study inclusion (*n*=10) | | | | | | | | | | | | | | | | | |
| Man | 50–64 | Septic arthritis | IgG lambda (36 g/L) | Multiple myeloma | Conv | 43 | | **0.32** | | **0.36** | | 26 | **0.38** | | | **0.26** | 0.053 |
| Man | 65–79 | Bacteremia without focus | IgG lambda (ND) | Multiple myeloma | Acute | 40 | | **0.04** | | **0.1** | | 33 | **0.08** | | | **0.06** | **0.01** |
| Man | 65–79 | Pneumonia | IgG kappa (ND), IgG lambda (ND) | Chronic lymphocytic leukemia | Acute | 12 | | **0.07** | | 0.53 | | 8.2 | 1.4 | | | 0.88 | 0.13 |
| Woman | 65–79 | Pneumonia | IgG kappa (0.5 g/L) | Multiple myeloma | Conv | **3.7** | | **0.3** | | **<0.10** | | **2.5** | **0.71** | | | 0.33 | **0.01** |
| Man | 65–79 | Meningitis, spondylodiscitis | IgG kappa (1 g/L) | Multiple myeloma | Acute | **3.2** | | **0.38** | | 1.5 | | **2.2** | **0.94** | | | 0.53 | **0.05** |
| Woman | 65–79 | Pneumonia | IgG kappa (0.5 g/L) | MALT lymphoma | Acute | **4.2** | | 1.7 | | 0.8 | | **2.6** | 1.2 | | | 1.6 | 0.077 |
| Man | 65–79 | Pneumonia | IgG kappa (29 g/L) | Multiple myeloma | Conv | 31 | | **0.36** | | 0.43 | | 19 | **0.58** | | | **0.09** | **0.03** |
| Man | 65–79 | Pneumonia | IgG kappa (0.5–1 g/L), IgG lambda (0.5–1 g/L), IgM lambda (<0.5 g/L), IgA (<0.5 g/L) | Chronic lymphocytic leukemia | Conv | 8.1 | | **0.51** | | **0.22** | | 5.8 | **0.79** | | | 0.87 | 0.15 |
| Man | 65–79 | Pneumonia | IgM kappa (5 g/L), IgG lambda (3 g/L), IgG kappa (0.5 g/L), IgA (1 g/L) | Waldenstrom macroglobulinemia | Acute | 6.9 | | 1.3 | | 5.8 | | 3.6 | 1.9 | | | 2.3 | 0.22 |
| Man | ≥80 | Pneumonia | IgG kappa (ND) | Multiple myeloma | Acute | 46 | | **0.36** | | 0.65 | | **1.5** | **0.74** | | | 0.79 | 0.05 |
| Newly diagnosed B-cell malignancy (*n*=7) | | | | | | | | | | | | | | | | | |
| Woman | 65–79 | Pneumonia | IgG lambda (40 g/L) | Multiple myeloma | Acute | | 53 | | **0.24** | | **0.13** | 42 | | **0.46** | **0.13** | | 0.52 |
| Woman | 65–79 | Pneumonia | IgM lambda (2 g/L) | Mantle cell lymphoma | Conv | | **5.2** | | **0.58** | | 2.6 | 3.6 | | **0.76** | 0.82 | | **0.01** |
| Man | 65–79 | Pneumonia | IgM lambda (10 g/L) | Waldenstrom macroglobulinemia | Conv | | **6.5** | | 1.5 | | 12 | 4.6 | | 1.7 | 0.26 | | **0.04** |
| Woman | 65–79 | Pneumonia | IgM lambda (9 g/L) | Waldenstrom macroglobulinemia | Conv | | 9.2 | | **0.47** | | 18 | 5.0 | | 2.0 | 0.89 | | 0.44 |
| Man | 65–79 | Meningitis, pneumonia, septic arthritis | IgG lambda (36 g/L) | Multiple myeloma | Acute | | 44 | | **0.14** | | **0.15** | 35 | | **0.26** | **0.04** | | **0.02** |
| Man | 65–79 | Pneumonia | IgA kappa (10 g/L) | Multiple myeloma | Acute | | 10 | | 15 | | 1.1 | 4.9 | | 2.6 | 2.1 | | 0.21 |
| Man | ≥80 | Pneumonia | IgG lambda (15 g/L) | Multiple myeloma | Acute | | 22 | | **0.63** | | **0.11** | 17 | | **0.41** | **0.09** | | 0.06 |
| Newly diagnosed monoclonal gammopathy of undetermined significance (*n*=12) | | | | | | | | | | | | | | | | | |
| Man | 18–49 | Pneumonia | IgG lambda (ND) | MGUS | Acute | | 11 | | 10 | | 0.42 | 8.7 | | 1.3 | 1.0 | | 0.16 |
| Man | 18–49 | Pneumonia | IgG kappa (10 g/L), IgA lambda | MGUS | Conv | | 13 | | **0.81** | | **0.25** | **1.7** | | 11 | 0.27 | | 0.10 |
| Man | 50–64 | Pneumonia | IgG lambda (1 g/L) | MGUS | Conv | | 14 | | 1.9 | | 0.93 | 9.6 | | 2.7 | 0.72 | | 0.31 |
| Man | 50–64 | Pneumonia | IgG lambda (6 g/L), IgG kappa (6 g/L) | MGUS | Conv | | 24 | | 1.0 | | 0.93 | 17 | | 1.6 | 0.58 | | 1.5 |
| Woman | 65–79 | Pneumonia | IgG lambda (0.5 g/L) | MGUS | Conv | | 11 | | 3.2 | | 1.0 | 5.9 | | 2.5 | 0.34 | | 0.24 |
| Man | 65–79 | Pneumonia | IgG kappa (7 g/L) | MGUS | Conv | | 14 | | 1.3 | | 1.8 | 9.1 | | 2.3 | 0.48 | | 0.53 |
| Man | 65–79 | Pneumonia | IgG lambda (11 g/L) | MGUS | Conv | | 17 | | **0.77** | | 0.39 | 3.4 | | 2.2 | 0.73 | | 10 |
| Woman | 65–79 | Pneumonia | IgM lambda (3 g/L) | MGUS | Acute | | **2.9** | | **0.52** | | 6.7 | **1.9** | | **0.15** | **0.14** | | **0.01** |
| Man | 65–79 | Pneumonia | IgA lambda (1–2 g/L) | MGUS | Conv | | 9.0 | | 2.9 | | 0.46 | 5.1 | | 2.7 | 0.63 | | 0.27 |
| Woman | 65–79 | Pneumonia | IgG lambda (2 g/L) | MGUS | Conv | | 8.6 | | **0.56** | | 1.3 | 6.0 | | **0.97** | 0.62 | | 0.10 |
| Man | ≥80 | Pneumonia | IgG lambda (2 g/L) | MGUS | Acute | | 10 | | 1.4 | | 0.61 | 6.2 | | 2.2 | 0.49 | | 0.54 |
| Man | ≥80 | Bacteremia without focus | IgM kappa (3 g/L) | MGUS | Conv | | **4.4** | | **0.17** | | 6.9 | 3.0 | | **0.89** | 0.31 | | 0.11 |
| Deceased or lost to follow-up (*n*=5) | | | | | | | | | | | | | | | | | |
| Woman | 50–64 | Pneumonia | IgG kappa (0.5 g/L) | Deceased before follow-up sampling | Acute | | 19 | | 5.3 | | 1.5 | 14 | | 3.3 | 1.3 | | 1.1 |
| Man | 65–79 | Pneumonia | IgG kappa (2–3 g/L) | Deceased before follow-up sampling | Acute | | 14 | | 4.2 | | 0.86 | 12 | | 1.4 | 0.47 | | 0.32 |
| Man | ≥80 | Pneumonia | IgG kappa (12 g/L) | Deceased before follow-up sampling | Acute | | 19 | | **0.47** | | 0.45 | 11 | | **0.56** | **0.13** | | 0.18 |
| Man | ≥80 | Bacteremia without focus | IgG kappa (ND) | Deceased before follow-up sampling | Acute | | 13 | | 1.2 | | 2.7 | 11 | | 1.6 | 0.76 | | 0.37 |
| Man | 50–64 | Pneumonia | IgG kappa (ND) | Lost to follow-up | Acute | | 15 | | 4.3 | | 2.9 | 11 | | 2.5 | 0.4 | | 0.14 |

Conv, convalescence; Ig, immunoglobulin; IPD, invasive pneumococcal disease; M protein, monoclonal protein; MALT lymphoma, mucosa-associated lymphoid tissue lymphoma; MGUS, monoclonal gammopathy of undetermined significance; ND, not determined. Patients with persistent M protein or individuals deceased or lost to follow-up are included in the table. Additionally, in seven patients, a transient M protein was normalized at follow-up. Two additional patients had previously known hematological malignancy without detected M protein.

^a^ Concentrations including M protein fraction. Numbers in bold indicate a value below the reference intervals: IgG 6.7–14.5 g/L; IgA 0.88–4.5 g/L; IgM 0.27–2.1 g/L; IgG1 2.8–8.0 g/L; IgG2 1.2–5.7 g/L; IgG3 0.24–1.3 g/L; IgG4 0.05–1.3 g/L.

**Supplementary Table S3.** Correlations between immunoglobulin (Ig) isotype concentrations, including M protein if any, and lymphocyte cell counts in patients during the convalescent phase, 2-4 months after an episode of invasive pneumococcal disease.

|  | Lymphocytes and lymphocyte sub-populations | | | | | |
| --- | --- | --- | --- | --- | --- | --- |
| Immunoglobulin | Lymphocytes | B cells | NK cells | Total T cells | Helper T cells | Cytotoxic T cells |
| IgG | r = .16 *p* = .20 | r = .097 p = .42 | r = .18 *p* = .15 | r = .19 *p* = .11 | r = .13 *p* = .28 | r = .10 *p* = .39 |
| IgA | r = .12 *p* = .33 | r = .033 *p* = .79 | r = .22 *p* = .07 | r = .18 *p* = .15 | r = .23 *p* = .06 | r = .11 *p* = .36 |
| IgM | r = -.17 *p* = .17 | r = .040 *p* = .74 | r = -.19 *p* = .12 | r = -.078 *p* = .52 | r = -.093 *p* = .44 | r = .010 *p* = .93 |
| IgG1 | r = .12 *p* = .34 | r = .074 *p* = .54 | r = .10 *p* = .39 | r = .11 *p* = .35 | r = .048 *p* = .69 | r = .057 *p* = .64 |
| IgG2 | r = .098 *p* = .42 | r = .13 *p* = .27 | r = .16 *p* = .19 | r = .13 *p* = .29 | r = .13 *p* = .27 | r = .11 *p* = .39 |
| IgG3 | r = .11 *p* = .37 | r = -.12 *p* = .32 | r = .13 *p* = .30 | r = .054 *p* = .66 | r = .016 *p* = .89 | r = .036 *p* = .77 |
| IgG4 | r = .15 *p* = .21 | r = -.063 *p* = .61 | **r = .36 *p* = .002** | r = .16 *p* = .17 | r = .088 *p* = .47 | r = .17 *p* = .15 |

**Supplementary Table S4.** Reference intervals for each type of immunoglobulin (Ig) isotype or IgG subclass.

| Ig | Reference interval (g/L) |
| --- | --- |
| IgG | 6.7–14.5 |
| IgA | 0.88–4.5 |
| IgM | 0.27–2.1 |
| IgG1 | 2.8–8.0 |
| IgG2 | 1.2–5.7 |
| IgG3 | 0.24–1.3 |
| IgG4 | 0.05–1.3 |

**Supplementary Table S5.** Reference intervals for lymphocytes and lymphocyte subpopulation cell counts.

| Lymphocyte population | Reference interval (x10^9^/L) |
| --- | --- |
| Lymphocytes | 1.0–2.8 |
| CD19+ B cells | 0.1–0.5 |
| CD3-CD19-CD56+CD16+ or CD3-CD19-CD56+CD16- NK cells | 0.09–0.6 |
| CD3+ T cells | 0.7–2.1 |
| CD3+CD4+ helper T cells | 0.3–1.4 |
| CD3+CD8+ cytotoxic T cells | 0.2–0.9 |


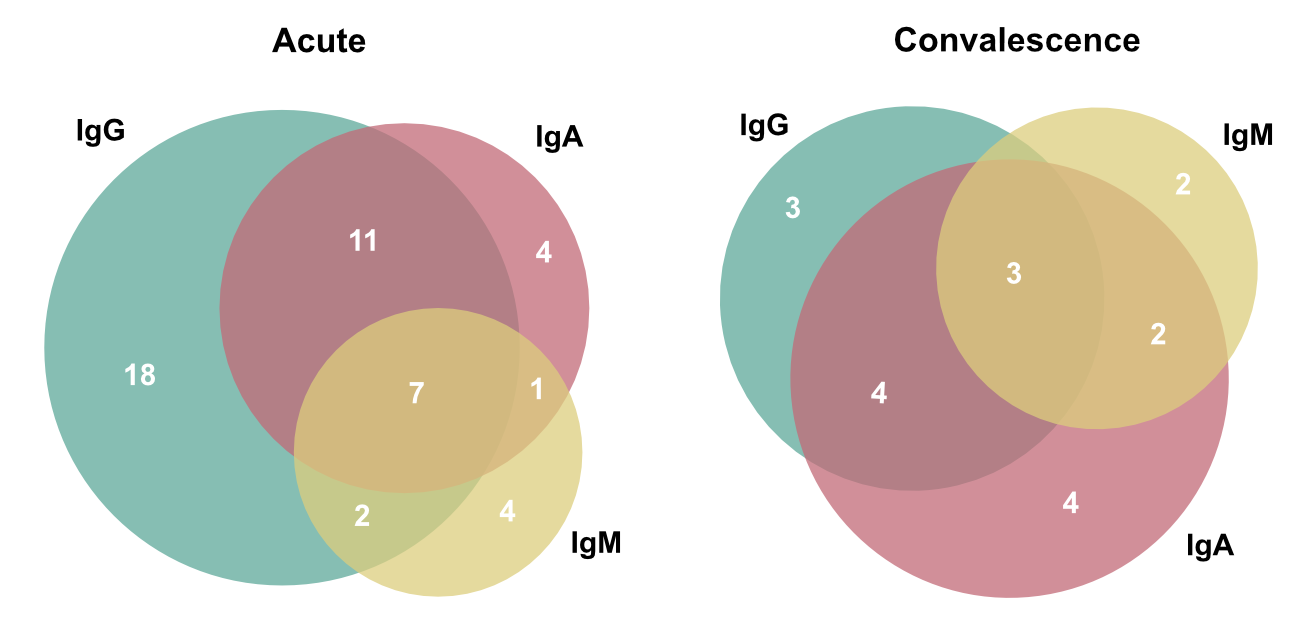


**Supplementary Figure S1.** Patients (n) with immunoglobulin levels below the reference intervals, during acute invasive pneumococcal infection, and in the convalescent phase 2-4 months later. M protein fractions are excluded. Each circle represents an Ig isotype, where the size of the circle indicates the proportion of patients. The numeric values indicate the number of patients with isolated or combined Ig levels below the reference interval.


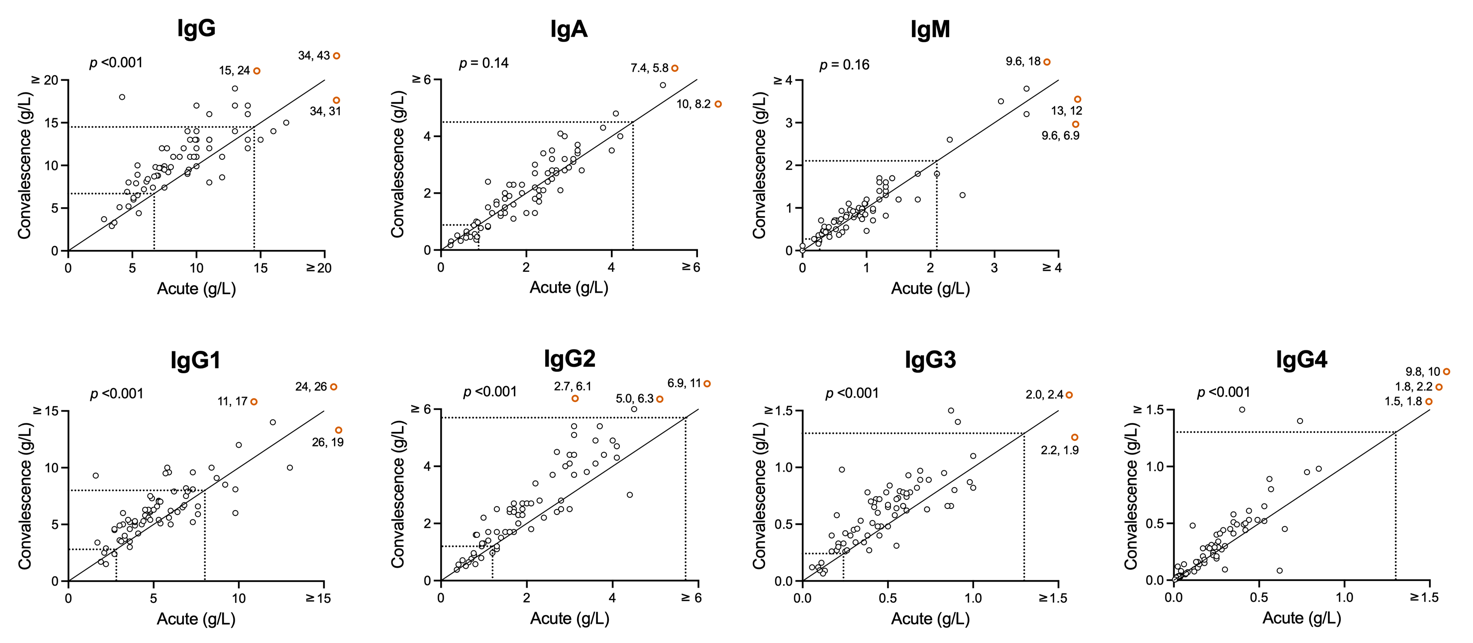


**Supplementary Figure S2.** Concentrations of paired serum immunoglobulin (Ig)G, IgA, IgM, and IgG subclasses 1–4 in invasive pneumococcal disease patients (*n*=73) during acute infection (x-axis) and in the convalescent phase (y-axis). M protein concentrations are included. Each individual is represented by one symbol. Dashed lines represent the lower and upper limit of the reference interval, respectively. Outliers are indicated by orange circles. If there is no change between acute infection and convalescence, symbols will follow the diagonal line.
